# Structure Based Thermostability Prediction Models for Protein Single Point Mutations with Machine Learning Tools

**DOI:** 10.1371/journal.pone.0138022

**Published:** 2015-09-11

**Authors:** Lei Jia, Ramya Yarlagadda, Charles C. Reed

**Affiliations:** 1 Amgen, Thousand Oaks, CA, United States of America; 2 Intrexon, Germantown, MD, United States of America; University of Michigan, UNITED STATES

## Abstract

Thermostability issue of protein point mutations is a common occurrence in protein engineering. An application which predicts the thermostability of mutants can be helpful for guiding decision making process in protein design via mutagenesis. An *in silico* point mutation scanning method is frequently used to find “hot spots” in proteins for focused mutagenesis. ProTherm (http://gibk26.bio.kyutech.ac.jp/jouhou/Protherm/protherm.html) is a public database that consists of thousands of protein mutants’ experimentally measured thermostability. Two data sets based on two differently measured thermostability properties of protein single point mutations, namely the unfolding free energy change (ddG) and melting temperature change (dTm) were obtained from this database. Folding free energy change calculation from Rosetta, structural information of the point mutations as well as amino acid physical properties were obtained for building thermostability prediction models with informatics modeling tools. Five supervised machine learning methods (support vector machine, random forests, artificial neural network, naïve Bayes classifier, K nearest neighbor) and partial least squares regression are used for building the prediction models. Binary and ternary classifications as well as regression models were built and evaluated. Data set redundancy and balancing, the reverse mutations technique, feature selection, and comparison to other published methods were discussed. Rosetta calculated folding free energy change ranked as the most influential features in all prediction models. Other descriptors also made significant contributions to increasing the accuracy of the prediction models.

## Introduction

Thermostability is a basic biophysical property of a protein. It connects tightly to protein expression, folding, activities and functions. Therefore, thermostability is one of the most important criteria to consider during protein engineering process. Commonly, very large libraries need to be built up for screening stable protein mutants. Such process is considerably expensive and time consuming. An *in silico* application which predicts the thermostability of mutants can be very helpful for guiding the decision making process in protein design via mutagenesis. An *in silico* point mutation scanning method is frequently used in finding the “hot spots” in proteins for focused mutations. In addition, *in silico* thermostability prediction can help to prioritize the large libraries into smaller focused ones in order to save time and cost.

Several protein thermostability prediction models have been developed [1–12]. High prediction accuracy as well as efficiency are still challenging in this field [13]. Note that most *in silico* methods can only claim to achieve moderate accuracy in protein stability prediction due to the complexity of many factors which contribute to protein stability.

ProTherm (http://gibk26.bio.kyutech.ac.jp/jouhou/Protherm/protherm.html) is a database which consists of thousands of protein mutants’ thermostability data. The data which were archived in ProTherm came from experimental measurements [14–19]. It's one of the most comprehensive protein thermostability database in the public domain. ProTherm is also the most popular resource for training thermostability prediction models which are reported in literature.

Rosetta is a state of art protein design tool featuring Monte Carlo simulated annealing sampling, which is capable to survey large conformation space of proteins and provides reasonable energy evaluation in short time. Time efficiency is considered a major advantage of Rosetta comparing to the classic force field based molecule mechanics. One very useful function in Rosetta is to calculate the stability influence of protein point mutations. The Rosetta DDG_monomer application uses a scoring function to calculate the preference between the wild type and mutant proteins. This score difference can be used as a descriptor to evaluate mutant thermostability [20]. Kortemme *et*. *al*. described the details of Rosetta DDG_monomer calculation [21].

Machine learning tools are widely used to provide artificial intelligence for building prediction models in many applications, including handwriting recognition, face detection, speaker identification, microarray expression data analysis, quantitative structure-activity relationship etc. Machine learning can be considered as a smart and efficient way for computer automatically making decisions on unseen data, based on learning from large and comprehensive training data. In this work, five supervised machine learning tools: support vector machine (SVM), random forests (RF), naïve Bayes classifier (NBC), K nearest neighbor (KNN), and artificial neural network (ANN) as well as one regression tool: partial least squares (PLS) were used for building protein thermostability predictions models with classification and regression analyses.

Quantitative structure—activity relationship (QSAR) models have been maturely applied to the small molecule drug discovery field [22, 23]. The predictive QSAR model is trained by a set of data with known activities. The derived model is then used to predict data with unknown activities. In this work, QSAR modeling has been attempted to protein design and engineering field for prediction of thermostability of protein single point mutations. Three key components of a QSAR modeling have been carefully designed and tested. They include a high quality and diversified data set to train the model, a biophysically meaningful and accurately derived descriptor set, as well as several powerful machine learning and regression algorithms. Binary prediction from the derived models achieved high thermostability prediction results. Ternary prediction resulted an acceptable accuracy. The regression case demonstrated that the introduction of simple physical properties of amino acids and structural properties can improve the performance of the prediction models.

## Methods

### Data set construction

Two data sets: unfolding free energy change (ddG) and melting temperature change (dTm) were constructed from ProTherm database. Each data point in the two data sets consists of a single point mutation from a protein which has at least an experimental structure available in Protein DataBank (PDB). To be relevant with physiological conditions, the data were collected between pH 6 and 8. Mutations were selected only in small and medium monomeric proteins with no more than 300 residues. Multiple data points under same conditions were averaged. For the ddG data set, we removed the mutations whose measured unfolding free energy changes are lower than -10 kcal/mol or greater than 10 kcal/mol, since these measurements are outliners and likely to have greater errors. We did not trim the dTm data set in order to keep it the similar size as the ddG set. For classification purpose, 2 ddG boundaries (-1, -0.5) kcal/mol and (0.5, 1) kcal/mol and 2 dTm boundaries (-3, -1)°C and (1, 3)°C were setup for defining stable, neutral, and unstable mutants (Table 1). Data sets are provided in the supporting information (S1 Table).

**Table 1:** Data set construction.

**Class**	**Measured ddG (kcal/mol)**	**Qualified Data**	**Class Structures / Total Structures** ^a^
Neutral	≥ -0.5 ≤ 0.5	323	37 / 51
Stable	≥ 1.0	69	20 / 51
Unstable	≤ -1.0	406	39 / 51
**Class**	**Measured dTm (°C)**	**Qualified Data**	**Class Structures / Total Structures** ^a^
Neutral	≥ -1.0 ≤ 1.0	223	47 / 82
Stable	≥ 3.0	141	40 / 82
Unstable	≤ -3.0	435	62 / 82

^a^ Class Structures: the number of PDB structures in each class. Total Structures: the total number of PDB structures in the data set. Some structures contain more than one class of mutations. The sum of structures in each class is greater than total structures in the data set.

### Descriptor set

The novel descriptor set being used for modeling contains three layers: 1. Physical property layer: changes of six canonical amino acid physical properties, 2. Structural property layer: two structural properties at the mutation site, and 3. Energetic layer: a high level folding energy change which was calculated by Rosetta (Table 2).

**Table 2:** The descriptor set used for modeling.

The change of basic physical properties of amino acids	Structure based	Energy
(1) Molecular weight; (2) Formal charge; (3) Hydrophobicity; (4) Aromaticity; (5) van der Waals volume; (6) Solvent accessible surface area	(1) Secondary structure where the mutation is located in experimental structure; (2) Percentage solvent accessible surface area (residue exposure)	(1) Rosetta DDG_monomer (mutant folding energy change)

The six canonical amino acid physical properties were quantified based on literature records [24–29]. They are molecular weight, formal charge, hydrophobicity, aromaticity, van der Waals volume, and solvent accessible surface area. The differences of these properties between the mutant and wild type amino acid were calculated and serve as descriptors for model building.

Two structural descriptors were obtained from ProTherm database: secondary structure which was observed from the experimental structure at the mutation site and percentage accessible surface area (ASA) of the wild type residue on protein level. The secondary structure feature was quantified as the following: alpha helix = 1, beta sheet = 2, coil = 3, turn = 4. The ASA was calculated by the following method: ASA (expressed in units of Å^2^ Accessibility (%)) is defined as the ASA of the residue at the mutation site (X) in its parent protein, computed with a program Analytical Surface Calculation (ASC) divided by the ASA of the residue in an extended tripeptide Ala-X-Ala conformation. The extended state ASA was calculated using ECEPP/2 algorithm with dihedral angles given by Oobatake and Ooi [30] and the van der Waals radius of atoms from Ooi et al. [31]. The values are Ala-110.2; Asp-144.1; Cys-140.4; Glu-174.7; Phe-200.7; Gly-78.7; His-181.9; Ile-185.0; Lys-205.7; Leu-183.1; Met-200.1; Asn-146.4; Pro-141.9; Gln-178.6; Arg-229.0; Ser-117.2; Thr-138.7; Val-153.7; Trp-240.5; Tyr-213.7 (the units are in Å^2^).

The mutants’ folding free energy changes were calculated by Rosetta DDG_monomer application with the high-level precision protocol (all atoms and backbone flexibility were considered). Fifty models for each of wild-type and mutant structures were generated by Rosetta. Please refer to reference [21] for details of DDG_monomer calculation. Protocol details are available in supporting information (S1 Text). Condor high throughput computing application was implemented to distribute thousands of Rosetta DDG_monomer calculations on computer cluster.

### Statistics algorithms

Different statistics algorithms have different advantages which are suitable for specific training and prediction purpose. To obtain high performance models for protein thermostability prediction purpose, we tested several statistics algorithms. Five supervised machine learning algorithms: support vector machine (SVM), random forests (RF), naïve Bayes classifier (NBC), K nearest neighbor (KNN), and artificial neural network (ANN) and a regression algorithm partial least squares (PLS) were applied to model building. All statistics algorithms were implemented with the caret package (v 6.0–35) [32] in the R project for statistical computing (v 3.1.1).

The method of support vector machines (SVM) was developed by Vladimir N. Vapnik at AT&T Bell Labs originally for discriminative classification to solve handwriting recognition problems [33]. The SVM model is trained by the data with known values. SVM trained model minimizes the generalization error by maximizing the margins from the hyper-plane to separate the positive and negative data. It’s capable to explore subtle patterns in a noisy data set by applying kernel functions and soft margins. SVM is capable for binary classification as well as multi-class classification and regression. This kernel based SVM is very powerful to make predictions by projecting the data to a higher dimensional feature space by a kernel function. However, using the kernel function may introduce overfitting problem.

The random forests (RF) method was developed by Leo Breiman of UC Berkeley [34]. It’s an ensemble classifier based on many decision tree models. RF can be applied for both classification and regression. Advantages of RF include the ability to establish interpretable models, accurate predictive results, resistant to overfitting problems, and fast training process.

Naïve Bayes classifier (NBC) is based on Bayes’ theorem [35]. It can only be applied for classification. NBC requires only a small amount of training data to estimate the parameters necessary for classification and can be scaled very well to very large data sets. NBC has a little difficulty with noisy or missing data.

K nearest neighbor (KNN) is a method for classifying objects based on closest training examples in the feature space (feature similarity clustering) [36]. It was originated from pattern recognition. KNN is one of the simplest machine learning algorithms. It can be applied for both classification and regression. The interpretable algorithm has simple implementation in which only one parameter—K needs to be tuned. One disadvantage of the method is that it’s computationally intensive.

Artificial neural network (ANN) is a mathematical model that is inspired by the structure and functional aspects of biological neural networks [37]. It can be used for both classification and regression. ANN is one commonly used artificial intelligent (AI) tool and able to learn from training data. When an element of the neural network fails, it can continue without any problem by its parallel nature. ANN requires a large diversity of training set in real-world operation.

Partial least squares (PLS) is one of the most commonly used regression tools in bioinformatics and cheminformatics [38]. It’s an extension of the multiple linear regression method. PLS can reduce many factors to a few latent factors thus avoids overfitting problem. Besides to its native regression application, PLS can also be used for classification.

### Model building

Three types of prediction models: binary classification for predicting stable and unstable mutants, ternary classification for predicting stable, neutral, and unstable mutants, as well as regression, were developed with the ddG and dTm data sets. Caret package automatically tuned the parameters in these models by grid search. We randomly selected 80% of data for training models in all statistics algorithms. Ten-fold cross validation was carried out by using the same training set. The accuracy of the 10-fold cross validation was used for evaluating different statistics algorithms.

To rigorously evaluate the modeling performance for generally predicting real data, a “blind” test was carried out for each model. The test set consisted of the rests of 20% data which were left from training data set selection as the “unseen” data. The accuracy from this test process was used for checking whether the models are overfitted. In a more rigorous test, we selected 20% data by protein for the “blind” test set. So, the test set contained proteins which do not present in the training set to avoid protein and amino acid residue position redundancy.

In the binary classification test case, in addition to prediction accuracy, receiver operating characteristics (ROC) curve was plotted and area under curve (AUC) value was calculated for each model to evaluate the prediction performance. In case of regression, coefficient of determination (R^2^) was calculated for evaluating the prediction performance.

### Hypothetical reverse mutations

The hypothetical reverse mutations technique is to build hypothetical reverse mutations based on the forward mutations that already exist in the data set. The unfolding free energy and melting temperature changes are state functions which are only governed by the properties at the beginning and end states. The values of state functions in a reverse mutation B-> A have a reversed sign comparing to those in the corresponding forward mutation A-> B [3, 4, 9]. This technique provides an additional validation step to check overfitting of the prediction models. We used the binary classification case which has the best prediction performance to test the models with reverse mutations. In the test, we built data sets based on reverse mutations from the original data sets. The signs of the following descriptors were reversed: delta_MW (molecular weight), delta_Chg (formal charge), delta_ARM (aromaticity), delta_Hydro (hydrophobicity), delta_VdwV (van der Waals volume), delta_SASA (solvent accessible surface area), and Rosetta calculated ddG since they are governed by state functions. The signs of SecSt (secondary structure at the mutation site) and ASA_pct (percentage of accessible surface area at the mutation site) were not reversed since they indicate the mutational location on a protein and thus are the same for both forward and reverse mutations. The reverse mutations data sets are provided in the supporting information (S1 Table).

We did 2 different tests based on the reverse mutations data. In the first test, we used models which were previously trained by forward mutations (80% data) to predict the reverse mutations “blind” test set (20% data). In the second test, we built new models by using a combination of forward and reverse mutations (80% data) for training. The combined forward and reverse mutations data set is more balanced than either of the 2 data sets. Then we tested the models with the combination of forward and reverse mutations “blind” test set (20% data).

### Feature selection

**Fig 1:**
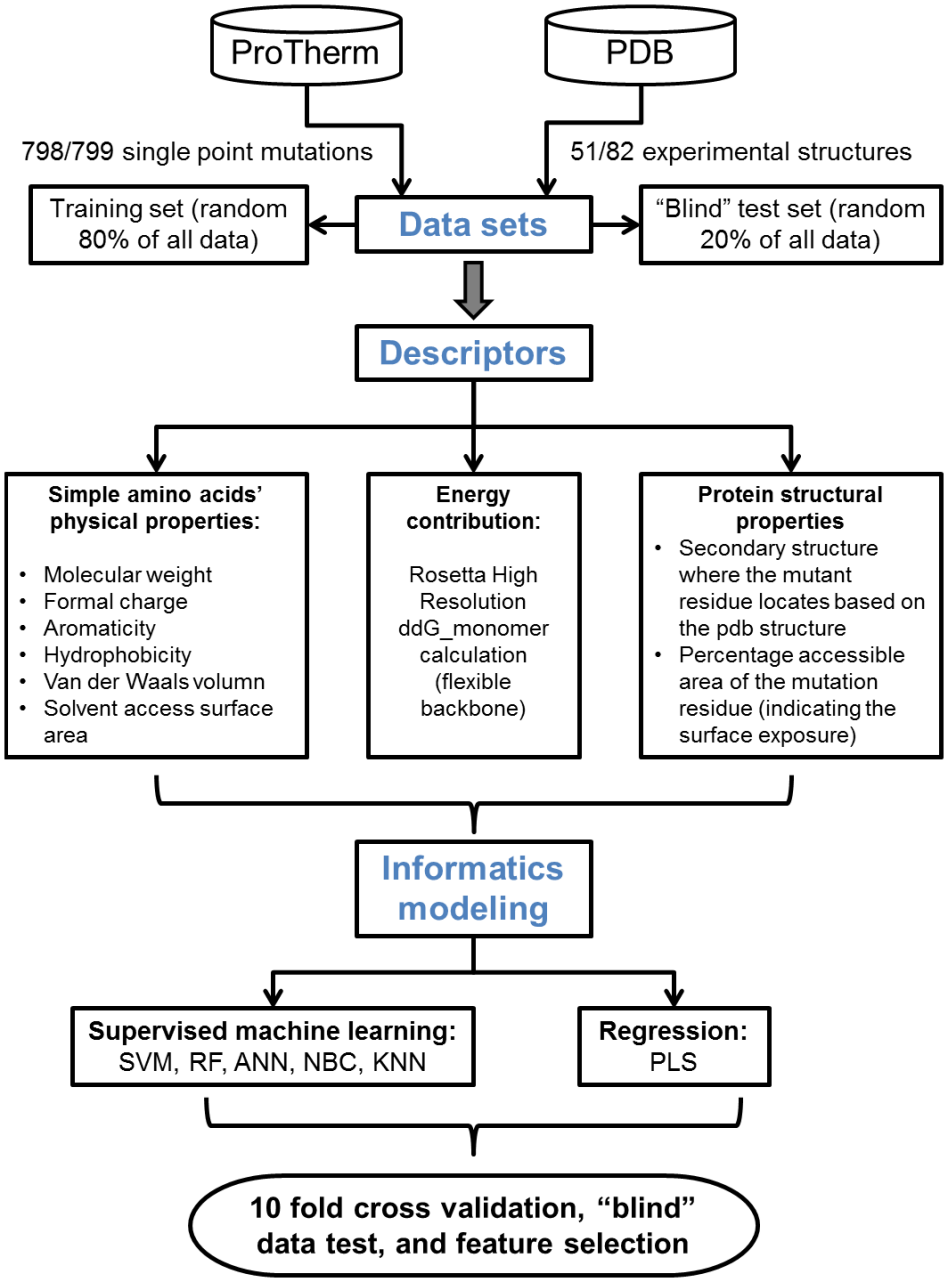
Workflow of model construction.

In order to connect statistics modeling results to the biophysical and structural information, feature selection was performed with recursive feature elimination (RFE) method from the caret package. RFE evaluates critical descriptors which contribute most to the prediction models. The high impact descriptors can help protein scientists to better design protein mutants and construct screening libraries based on understanding the protein thermostability on structural and biophysical levels. Fig 1 shows the overall workflow of model construction process.

## Results and Discussion

### Data set construction

**Fig 2:**
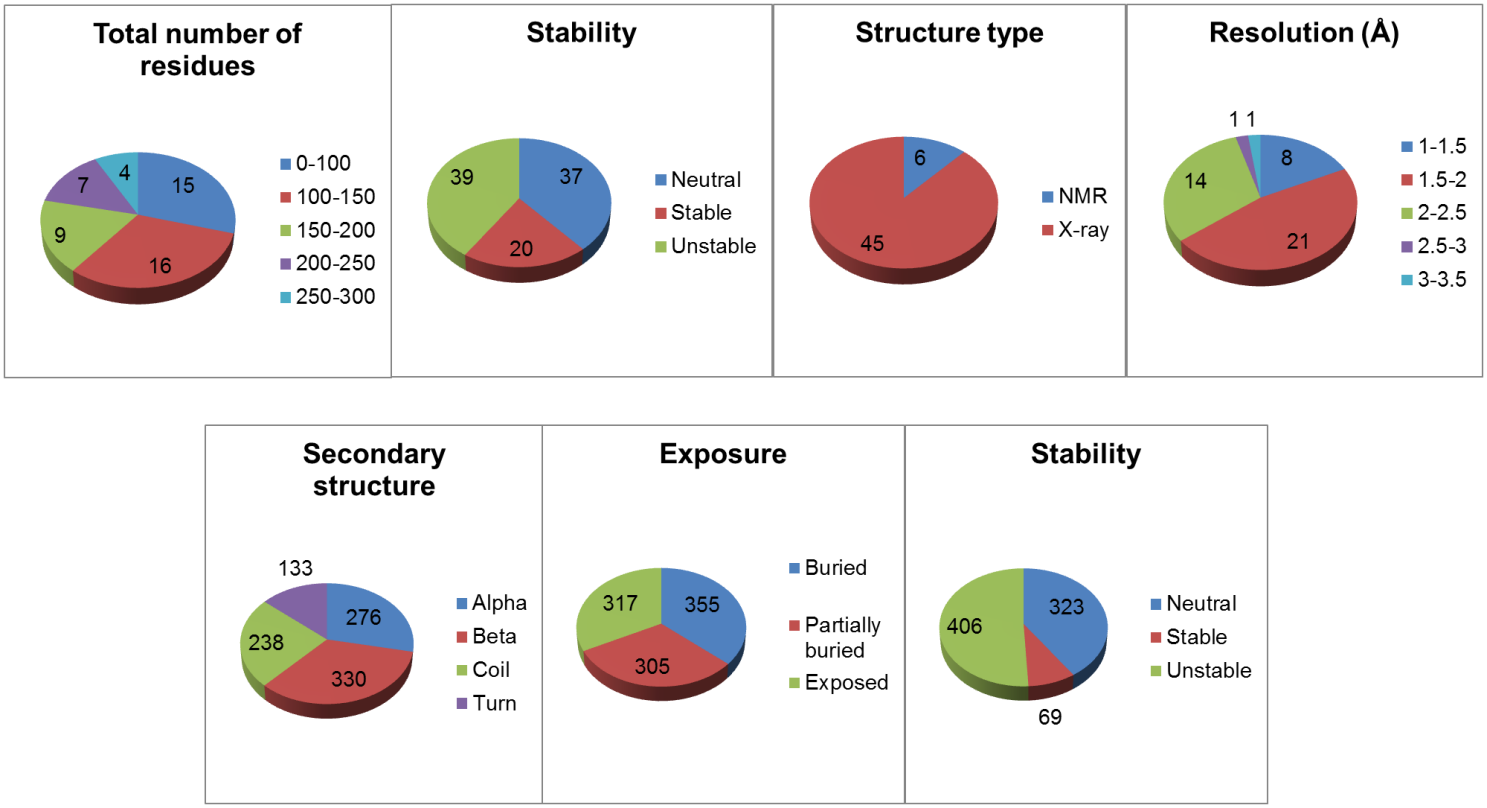
The diversity of protein structures (top 4 charts) and point mutation residues (bottom 3 charts) in the ddG data set. The sum of all proteins in the “Stability” pie chart is greater than the total number of proteins in Table 1, because some proteins have multiple mutations in different stability categories i.e. stable, neutral, or unstable. The “Resolution” chart only has data from proteins which have crystal structures. An NMR structure doesn’t have a resolution.

**Fig 3:**
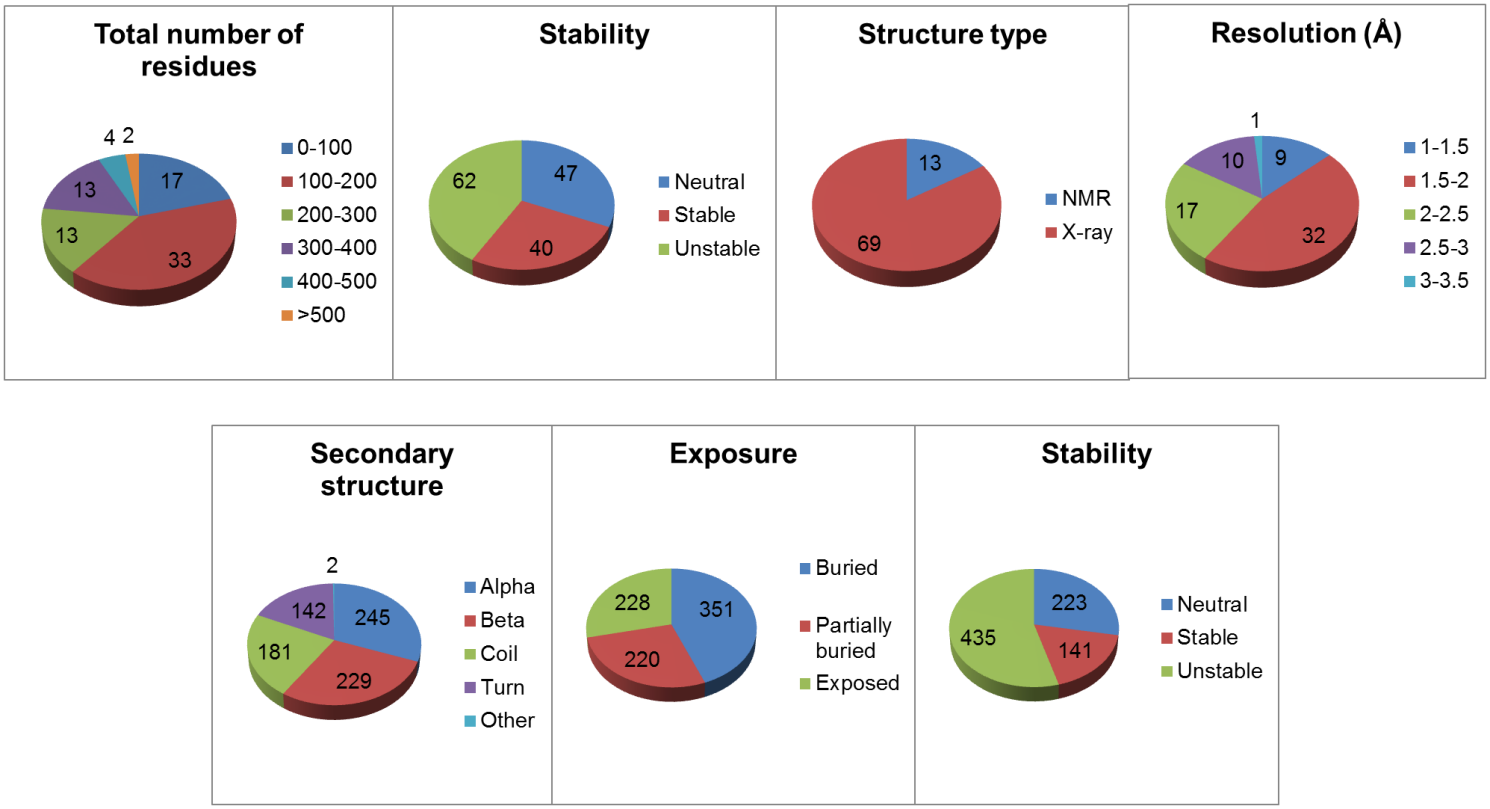
The diversity of protein structures (top 4 charts) and point mutation residues (bottom 3 charts) in the dTm data set. The sum of all proteins in the “Stability” pie chart is greater than the total number of proteins in Table 1, because some proteins have multiple mutations in different stability categories i.e. stable, neutral, or unstable. The “Resolution” chart only has data from proteins which have crystal structures. An NMR structure doesn’t have a resolution.

**Fig 4:**
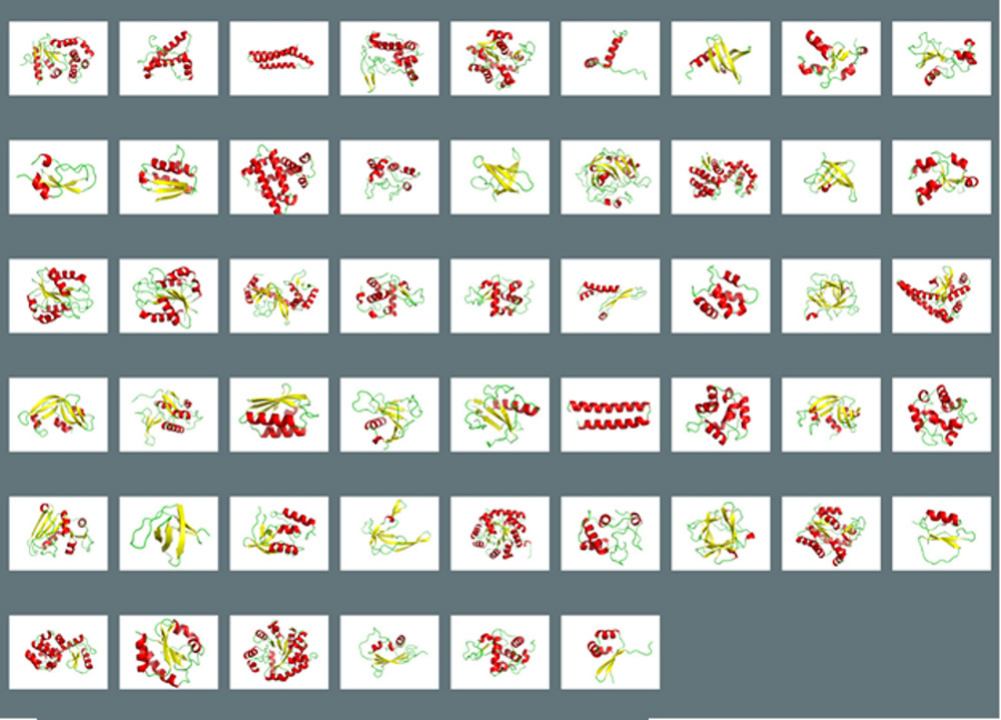
Protein structures in ddG prediction models. Alpha helixes are in red, beta sheets are in yellow, turns are in green. A diverse set of proteins with a mixture of different secondary structures were obtained.

**Fig 5:**
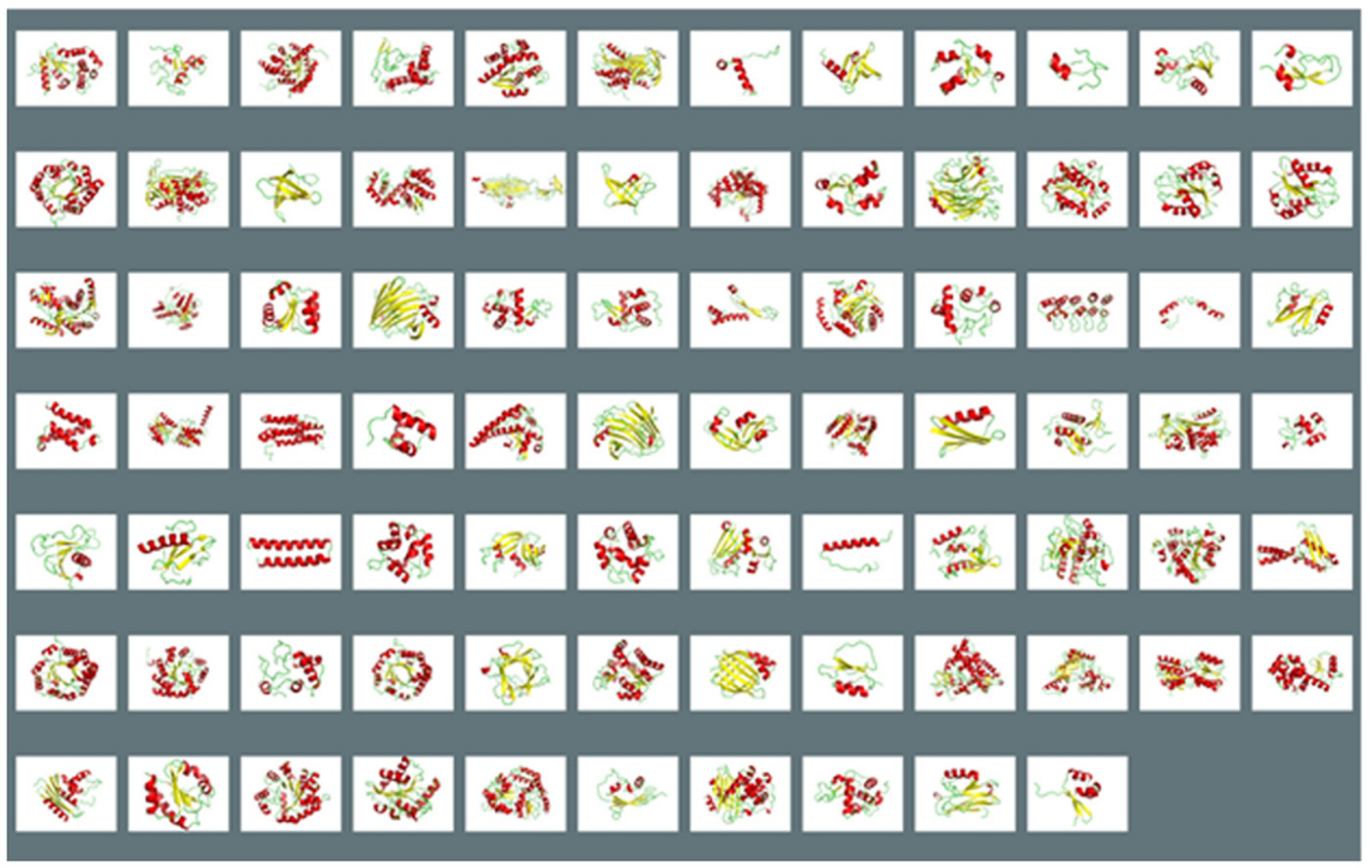
Protein structures in dTm prediction models. Alpha helixes are in red, beta sheets are in yellow, turns are in green. A diverse set of proteins with a mixture of different secondary structures were obtained.

The unfolding free energy change (ddG) data set contains 798 mutants from 51 different protein structures. The more challenging melting temperature change (dTm) data set contains 799 mutants from 82 different protein structures (Table 1). Both data sets contain a diversified structural and mutational data (Figs 2 and 3). Protein structures (Figs 4 and 5) in these thermostability prediction models contain a wide variety of protein classes as defined in Structural Classification of Proteins database (SCOP) [39]. A diversified data set is very important to establish generalized prediction models.

### Training and testing classification and regression models

**Fig 6:**
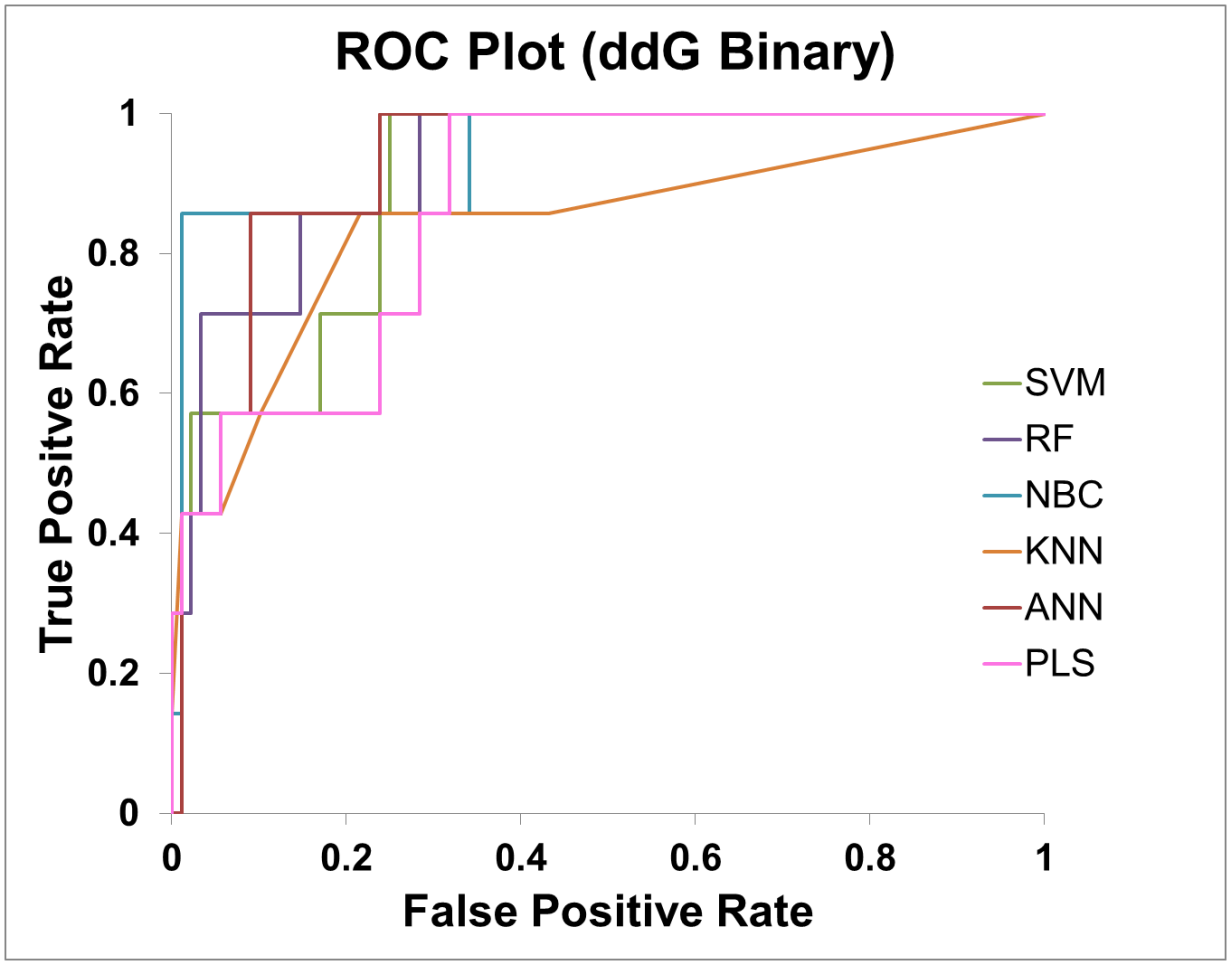
The ROC curves of ddG binary classification models for predicting the “unseen” data.

**Fig 7:**
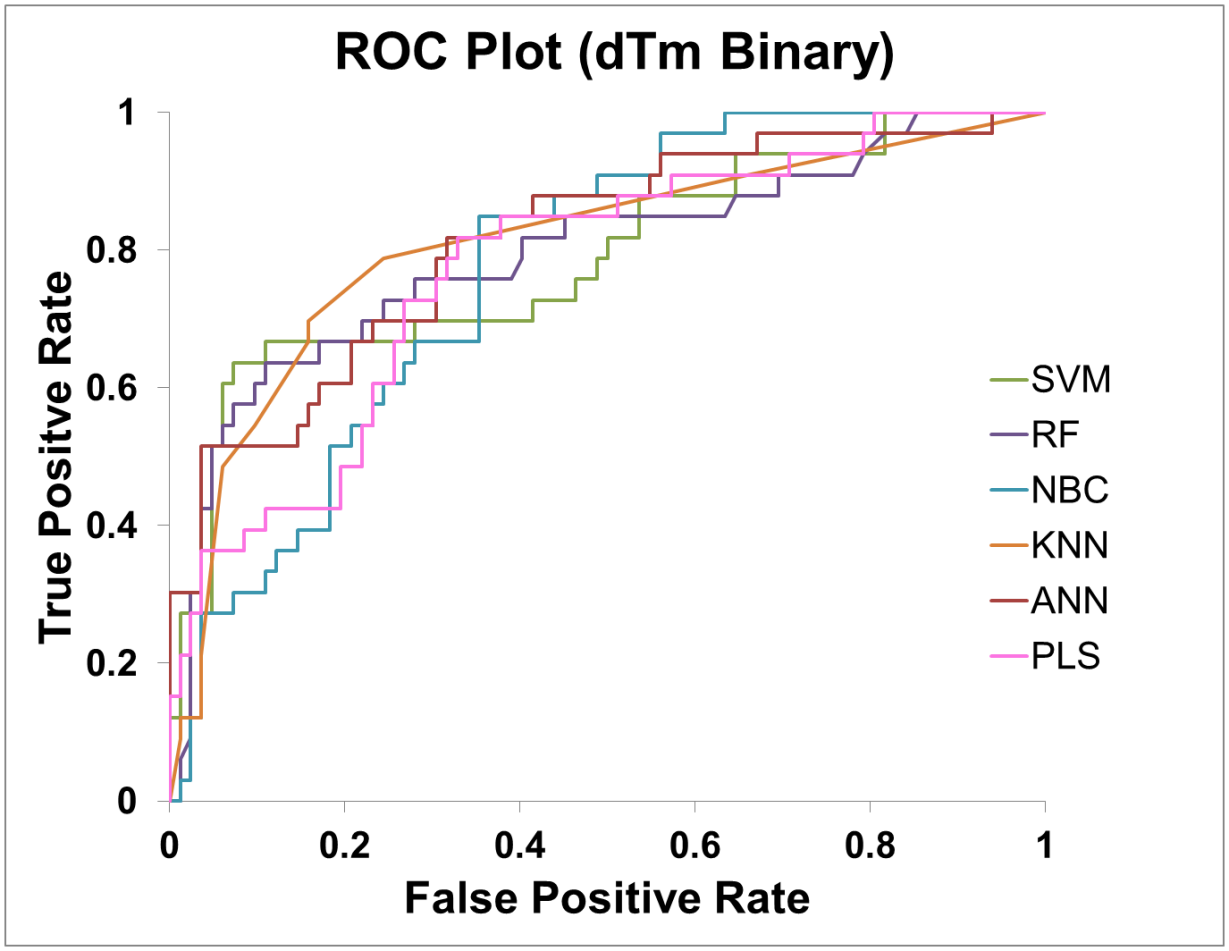
The ROC curves of dTm binary classification models for predicting the “unseen” data.

In binary classification of the ddG data set, the best prediction performance was obtained from SVM and ANN with 90% accuracy in 10-fold cross validation (CV) (Table 3). For the more challenging melting temperature prediction, RF performed the best with 85% accuracy in 10-fold CV. (Table 4). In all top performance models, the accuracy in the “blind” test is better or comparable to that in cross validation. Thus, these models were not overfitted based on this testing. The ROC curves of the “blind” test are shown in Figs 6 and 7. The AUC values of the “blind” test are shown in Tables 3 and 4.

**Table 3:** ddG binary classification.

Methods	SVM	RF	NBC	KNN	ANN	PLS
CV accuracy	***90%***	88%	85%	87%	***90%***	86%
Test accuracy	95%	93%	89%	94%	92%	95%
Test AUC	0.90	0.92	0.94	0.84	0.93	0.87

**Table 4:** dTm binary classification.

Methods	SVM	RF	NBC	KNN	ANN	PLS
CV accuracy	83%	***85%***	78%	82%	82%	81%
Test accuracy	82%	83%	73%	81%	76%	77%
Test AUC	0.79	0.79	0.77	0.81	0.81	0.77

Ternary classification is much harder than the binary classification due to the one additional class. In order to be counted as a corrected prediction for accuracy calculation, all 3 classes need to be predicted correctly. In the case of ddG prediction, SVM, RF, and ANN performed equally well with 69% accuracy in 10-fold CV (Table 5). For predicting the melting temperature change, SVM resulted 63% accuracy in 10-fold CV (Table 6). The accuracy in the “blind” test is comparable to that in cross validation in these top performed models except for ANN in ddG ternary classification. This indicates those models are not overfitted. ANN in ddG ternary classification may be considered to be slightly overfitted since the “blind” test accuracy is 4% lower than that of the cross validation.

**Table 5:** ddG ternary classification.

Methods	SVM	RF	NBC	KNN	ANN	PLS
CV accuracy	***69%***	***69%***	64%	65%	***69%***	65%
Test accuracy	68%	71%	63%	61%	65%	64%

**Table 6:** dTm ternary classification.

Methods	SVM	RF	NBC	KNN	ANN	PLS
CV accuracy	***63%***	62%	59%	62%	62%	61%
Test accuracy	63%	67%	66%	66%	63%	67%

In ddG regression models, RF gave the best 0.43 coefficient of determination (R^2^) in 10-fold CV (Table 7). In dTm regression models, SVM and RF gave the best 0.31 R^2^ in 10 fold CV (Table 8). The regression modeling process demonstrated an improvement of models by including amino acids’ simple physical properties and structural descriptors comparing to just using the Rosetta folding energy change calculation alone. Please see the Feature Selection section for details.

**Table 7:** ddG regression.

Methods	SVM	RF	KNN	ANN	PLS
CV R^2^ value	0.35	***0*.*43***	0.31	0.24	0.33
Test R^2^ value	0.41	0.43	0.36	0.15	0.35

**Table 8:** dTm regression.

Methods	SVM	RF	KNN	ANN	PLS
CV R^2^ value	***0*.*31***	***0*.*31***	0.30	0.15	0.25
Test R^2^ value	0.25	0.27	0.24	0.10	0.22

### Comparison of statistics algorithms

In our 6 different predictions, SVM performed the best in 4 predictions (ddG binary and ternary classification, dTm ternary classification and regression). RF also performed the best in 4 predictions (ddG ternary classification and regression, dTm binary classification and regression). These 2 machine learning algorithms feature finding subtle patterns in complex data. RF, if it is applied properly, tends to be less prone to overfitting than other methods. SVM can also minimize overfitting. We used a radial kernel function with SVM in this study. Radial kernel function is less overfitting comparing to some high degree polynomial kernel functions. SVM and RF methods are also very popular for protein thermostability predictions which are reported in literature.

### Feature selection and improvements from the novel descriptor set

To connect the statistics prediction with biophysical and structural information of the models, feature selection was carried out with recursive feature elimination (RFE) in the caret package. RFE ranks the importance of the descriptors by comparing their weight contribution to a model. For all types of predictions, Rosetta calculated ddG always ranked the highest among all nine descriptors. It demonstrated the importance of the Rosetta calculated energy term in these prediction models. Rosetta energy term provided a comprehensive representation and detailed molecular interactions (if using each individual Rosetta energy term) of the stability change. The percentage accessible surface area and hydrophobicity also played important roles in the thermostability prediction models (Table 9). Mutating surface residues and buried residues influenced the protein thermostability significantly. Changing residue polarity was also found to be important. This conclusion is consistent with commonly accepted rules that increasing protein surface polarity and increasing protein core hydrophobic packing can generally improve the protein thermostability.

**Table 9:** Top ranked features (descriptors) which are selected by RFE. Numbers depict the importance ranking of the descriptors in each model.

Features	ddG Bin	ddG Ter	ddG Reg	dTm Bin	dTm Ter	dTm Reg
Rosetta ddG	1	1	1	1	1	1
Percentage accessible surface area	2	2	2	3	2	2
Hydrophobicity	3		3	2	3	4
Formal charge				4		
Molecular weight						3
van der Waals volume			4			

We used the regression modeling to demonstrate an improvement of models by including amino acids’ simple physical properties and structural descriptors comparing to just using the Rosetta folding energy change calculation alone. We compared the R^2^ values between the best machine learning models (RF models in ddG and dTm) by using all descriptors and a simple linear regression model by using only Rosetta derived ddG (a single feature). In this comparison, the “blind” test results were used. For ddG prediction, the best RF model gave 0.43 test R^2^ (Table 7). Using only Rosetta derived ddG to perform a simple linear regression resulted a R^2^ of only 0.22. By including the amino acids’ simple physical properties and structural information as descriptors, the coefficient of determination increased 0.21 unit. For dTm prediction, RF gave 0.27 test R^2^ (Table 8), which was 0.06 unit higher than the R^2^ (0.21) from a simple linear regression by using the Rosetta calculated ddG as the only feature.

Despite of intrinsic overlapping between descriptors that include amino acids’ physical properties / local structure of the mutation and the Rosetta ddG which is a scoring function consists of multiple structural evaluations, the additional descriptors on top of Rosetta scoring function provide detail and straight forward description of “what” and “where” the single point mutation occurs in the target protein. Furthermore, using simple amino acids' physical properties has the following advantages: these properties are "solid" features which can be derived in high accuracy; they can be very helpful to interpret the model straight forwardly. These descriptors represent "what" is the mutation. The structural properties describe "where" the mutation locates, on the surface of the protein or buried inside the protein as well as the local secondary structure.

### Non-redundant testing for ddG classifications

In a more rigorous test, we constructed non-redundant training and test data sets for ddG binary and ternary classifications. We randomly selected 80% data by protein as the training data set. The rest 20% data was for “blind” testing. The test set contained proteins which do not present in the training set to avoid protein and amino acid residue position redundancy. We applied the exactly same training and testing procedure.

Comparing to the previous “redundant” test set results (Tables 3 and 5), the “non-redundant” (at both protein and amino acid residue position levels) test set results (Tables 10 and 11) showed that for ddG binary classifications, the prediction performance slightly decreased. In the “redundant” test, the best accuracy was 95% (SVM and PLS) and the best AUC was 0.94 (NBC). In the “non-redundant” test, the best accuracy was 92% (RF) and the best AUC was 0.84 (SVM). This indicated that some binary classification models are slightly overfitted on the protein or position level. For the ternary classification, the prediction performance was comparable or even increased. In the “redundant” test, the best accuracy was 71% (RF). In the “non-redundant” test, the best accuracy was 78% (RF). To explain the increased performance in the ternary classification case, we thought that the previous “redundant” test was probably not overfitted and even underperformed due to the test set selection (randomly picked 20% data). The “non-redundant” test set selection was targeted to a certain group of protein.

**Table 10:** Non-redundant ddG binary classification (training and test data sets were selected by protein).

Methods	SVM	RF	NBC	KNN	ANN	PLS
CV accuracy	88%	89%	87%	88%	88%	87%
Test accuracy	90%	***92%***	82%	90%	91%	91%
Test AUC	***0*.*84***	0.82	0.65	0.35	0.81	0.63

**Table 11:** Non-redundant ddG ternary classification (training and test data sets were selected by protein).

Methods	SVM	RF	NBC	KNN	ANN	PLS
CV accuracy	67%	69%	62%	63%	67%	61%
Test accuracy	70%	***78%***	76%	68%	73%	72%

### Hypothetical reverse mutations to detect overfitting and unbalanced data sets

The hypothetical reverse mutations technique is a very good way to increase sampling of the data set without adding new proteins. It provides an additional validation step to detect possible overfitting in the prediction models. For many machine learning methods, overfitting can be encountered due to close correlation between training and testing data sets. We applied hypothetical reverse mutations to our binary models which have the best prediction performance. Comparison between test results by using forward and reverse mutations is shown in Tables 12 and 13.

**Table 12:** ddG binary classification with forward and reverse mutations.

Methods	SVM	RF	NBC	KNN	ANN	PLS
Forward mutations training and test						
CV accuracy	90%	88%	85%	87%	90%	86%
Test AUC	0.90	0.92	0.94	0.84	0.93	0.87
Forward mutations training and reverse mutations test						
Test AUC	0.70	0.76	0.66	0.79	0.80	0.80
Forward + reverse mutations training, forward + reverse mutations test						
Test AUC	0.96	0.96	0.90	0.92	0.95	0.93

**Table 13:** dTm binary classification with forward and reverse mutations.

Methods	SVM	RF	NBC	KNN	ANN	PLS
Forward mutations training and test						
CV accuracy	83%	85%	78%	82%	82%	81%
Test AUC	0.79	0.79	0.77	0.81	0.81	0.77
Forward mutations training and reverse mutations test						
Test AUC	0.73	0.72	0.61	0.70	0.77	0.76
Forward + reverse mutations training, forward + reverse mutations test						
Test AUC	0.85	0.85	0.75	0.81	0.85	0.84

In the first test, we used the same model being trained by forward mutations with experimental data. We then compared the test AUC value between predicting forward mutations and predicting reverse mutations in the test set (since the training models are the same in this case, the CV accuracy is the same too). In this case, the test AUC values of most models in the reverse mutations test were lower than those in the forward mutations test. The difference of test AUC values was greater than 0.1 in the following models: SVM, RF, NBC, and ANN in the ddG binary classification, and NBC and KNN in the dTm binary classification. This indicated that those models may be overfitted. The forward mutations training data had more unstable mutations than stable mutations. When using models being trained by forward mutations data, the prediction had a tendency to predict unstable mutations. Therefore, when predicting the reverse mutations test set, where the majority data were stable mutations, the performance decreased in the overfitted models.

In the second test, we mixed forward and reverse mutations in training and test data set to balance the data sets. The performance of prediction models by using a balanced data set was better than either forward or reverse mutations data set with most algorithms. This demonstrated that a balanced training data set can increase the generalization performance of prediction models. However, it is not easy to obtain a balanced protein thermostability data set from experiments since most mutations on a wild type protein are unstable. Therefore, using reverse mutations technique to balance the data set can be useful.

### Comparison to other methods

To evaluate the performance of our models (ddG prediction), we compared them with 11 other methods which were previously reported in literature. Due to the limitation of direct access to these methods for training and testing with our data set, we were not able to obtain performance of these other methods by using our own data set. However, we managed to obtain the performance of these other methods from publications. MUpro, I-Mutant 2.0, LSE, PROTS, and PROTS_RF performance was obtained from Li, et al. [3]. FoldX and EGAD performance was obtained from Potapov et al. [11] PoPMuSiC-2.0, Prethermut, ProMaya, and ELASPIC performance was obtained from Berliner et al. [12]. One best performed model from each of our ddG binary and ternary classification as well as regression models was included in the comparison. Cross validation accuracy and correlation coefficient (Pearson’s r) were used for comparison except for FoldX, and EGAD. In FoldX and EGAD, the accuracy and the r value were obtained by using the directly calculated ddG of data sets containing 1200 and 1065 mutants, respectively. The accuracy and the r value in MUpro, I-Mutant 2.0 LSE, PROTS, and PROTS_RF model were obtained from a 5-fold cross validation, while we ran a 10-fold cross validation with our models. PoPMuSiC-2.0, Prethermut, ProMaya, and ELASPIC were evaluated with a 20-fold cross validation. The Potapov_09 (Core) data set [11] which contains 2104 mutations in 79 proteins was used for training and testing with these 4 methods. We converted our coefficient of determination (R^2^) to Pearson’s r by taking a squared root operation.

Comparing between the regression cases (Table 14), our RF model outperformed others but not Prothermut, ProMaya, and ELASPIC. The Potapov_09 (Core) data set which was used to evaluate these higher performance models is more than 3 times the size as our cross validation set. The ideal comparison is to use the same benchmark data set. Unfortunately, we couldn’t apply the Potapov_09 (Core) data set to our models in this paper due to technical reasons. Our binary classification has a better accuracy than other methods. But our binary classification has a ddG gap being applied in the data set and neutral mutants were excluded. In addition, our best binary classification model may be overfitted. The accuracy of our ternary classification is comparable to the accuracy of the other methods. It is close to the average of the accuracy from the other methods.

**Table 14:** Comparison of ddG prediction performance.

Methods	Accuracy	r
Binary classification with SVM model in this paper ^a^	0.90	
Ternary classification with RF model in this paper ^a^	0.69	
Regression with RF model in this paper ^a^		0.66
MUpro ^b^	0.81	0.48
I-Mutant 2.0 ^b^	0.78	0.54
LSE ^b^	0.61	0.16
FoldX ^d^	0.71	0.50
EGAD ^d^	0.73	0.60
PROTS (Structure based) ^b^	0.79	0.40
PROTS_RF (Structure based) ^b^	0.80	0.63
PoPMuSiC-2.0 ^c^		0.62
Prethemut ^c^		0.72
ProMaya ^c^		0.74
ELASPIC ^c^		0.77

^a^ 10-fold cross validation

^b^ 5-fold cross validation

^c^ 20-fold cross validation

^d^ Direct prediction.

### Unfolding free energy change (ddG) and melting temperature change (dTm) predictions

Two data sets, namely the unfolding free energy change (ddG) and melting temperature change (dTm) data sets, were constructed and used for training thermostability prediction models. The ddG data set was constructed based on experimental measurements of protein unfolding free energy change data. In all three types of our predictions, ddG predictions always better performed than dTm predictions. In addition, majority of protein thermostability predictions in literature are ddG models. And dTm predictions are rarely reported. Melting temperature is a direct indication of protein thermostability. Protein scientists often use melting temperature to describe the stability of a given protein. Therefore, the dTm prediction would be better accepted by protein scientists.

As discussed in an article by Becktel et al [40], under certain approximations (“ddG must be small and Tm of the wild type protein must not be too close to T_s_”, which is the temperature at which the wild type protein’s entropy change dS is zero), dTm = ddG/dS _g_ * where dS _g_ * is the entropy change of the mutant protein at dG = 0 or the slope of the mutant protein’s stability curve at Tm (dG = 0). In our prediction models, the dS _g_ * factor was not directly considered by any descriptors. In addition, our dTm data set has a larger dynamic range comparing to the ddG data set. We removed the mutations whose measured unfolding free energy changes are lower than -10 kcal/mol or greater than 10 kcal/mol when constructing the ddG data set. But we did not trim the dTm data set the same way in order to keep ddG and dTm sets in the comparable size. So there are more outliners in the dTm data set than the ddG set. And the dTm data set may deviate from the Becktel et al approximation (ddG must be small). Other unclear factors, like measurement consistency in data set etc, may also affect dTm prediction.

### Rosetta improvements

Rosetta calculated ddG was the single most important contributor to all prediction models as we found in feature selection. This property is a comprehensive feature which covers structural and “energetical” information. The accuracy of this property can significantly influence the overall performance of the prediction models. The ddG term being calculated by Rosetta is a scoring function, which considers certain intra-molecular interactions such as steric effect, hydrogen bonds etc. However, Rosetta is not set up by using a full atom based molecular mechanics force field. Therefore, the Rosetta ddG term is not actually in an energy form. It represents a “fitness” of certain molecular structures in relationship to a control system (a wild type protein in the mutant thermostability prediction case). The Rosetta ddG is only meaningful for relative comparison. Energy properties from molecular mechanics calculations such as MM-PBSA can be used as a surrogate to Rosetta ddG. When sampling the same conformational space, molecular mechanics energy is often more accurate than Rosetta ddG. However, Rosetta is efficient in surveying conformational space of proteins with consideration of both side chain and backbone flexibility. In comparing to other machine learning based protein thermostability prediction methods, this approach has a compatible but not the best performance. Increasing the accuracy of Rosetta ddG would be a readily approach to improving the prediction accuracy of the models in this paper.

### Portable QSAR modeling

Three components in a typical QSAR model: a data set, a descriptor set, and a statistical algorithm make it a portable method. The QSAR methodology was originally developed for predicting biological activities of small molecules for drug discovery [41–43]. In a frequently used application, a data set of chemical compounds’ concentration at half inhibition activity (IC50) are used to train a predictive model. The small molecules’ structural and physical properties are used as the descriptor set. A regression algorithm such as multiple linear regression or partial least squares is used to establish the model. QSAR modeling is also commonly used to predict drug compounds’ absorption, distribution, metabolism, excretion and toxicity (ADMET). In the current work, the same QSAR modeling framework was adopted to predict thermostability of a more complicated biological entity—proteins. We obtained a training data set, which contains experimental measured protein thermostability data. The molecular descriptors were tailored to this specific problem: protein thermostability. And several statistics algorithms were used to establish predictive models and compared to each other in terms of performance. Such QSAR model can be applied to a broader scope. As long as a uniformly and accurately measured training data set can be obtained; a meaningful set of properties as descriptors can be derived; and a statistics algorithm which is capable to provide inferential analysis of the data based on the descriptor set can be identified, a predictive QSAR model can be established. QSAR methods to predict activities and functions of biological system exist elsewhere [44]. The flexible QSAR models do not rely on any understanding of the physical mechanisms of the system. However, suitable physical properties can be involved through descriptors [45].

## Conclusion

There are lots of challenges to apply machine learning based QSAR models in protein engineering. Proteins have more complicated structures. Protein folding is still a puzzle which is not completed solved. On the other hand, proteins have more activities and functions to be predicted than those in small molecule field. Yet descriptor sets are under development. This work started from simple: applying amino acids' simple physical properties and structural information in addition to the Rosetta folding energy calculation.

Several thermostability prediction models were built for protein single point mutations. These models can be useful to prioritize protein engineering design libraries to save time and cost. To enhance the prediction performance, state of the art QSAR modeling was applied. It involved high quality and diversified data sets, an accurately derived and scientifically meaningful descriptor set, and several powerful machine learning and regression algorithms.

The models being reported here are limited to predicting thermostability of a single point mutation. Multiple point mutations are often involved in protein engineering. Predicting thermostability of multiple point mutations are possible but with great challenges. Rosetta folding energy calculation can handle mutants with more than one point mutation. The simple amino acid biophysical properties change and structural descriptors need to be synergized to each other to treat multiple point mutations properly. In addition, the experimental data with multiple point mutations are more complicated and noisy than those in the single point mutation case. This can also affect overall prediction quality of the multiple point mutation models. Further developing thermostability prediction models which consider multiple point mutations are under consideration. In addition, more sophisticated descriptors need to be developed for broader applications in the protein engineering field.

## Supporting Information

S1 Table Data sets for training and test in ddG and dTm binary and ternary classifications as well as regression. Data sets of reverse mutations and non-redundant construction are also included.
(XLSX)

S1 Text Rosetta protocol for DDG_monomer calculation. (DOCX)
