# Supplementary material for: Structure Based Thermostability Prediction Models for Protein Single Point Mutations with Machine Learning Tools
Source: PLoS One. 2015 Sep 11;10(9):e0138022. doi: 10.1371/journal.pone.0138022 (PMC4567301; doi:10.1371/journal.pone.0138022)
Supplement: S1 Text — (DOCX) [file pone.0138022.s002.docx]

**Rosetta protocol for DDG_monomer calculation**

DDG calculation requires the input structure to be pre-minimized by Minimize_with_cst protocol even the structure is derived from crystallography. This relaxation run also generates the distance restraints between C-alpha pairs within 9Å. A sample set of parameters for Minimize_with_cst calculation is listed below:

-in:file:l pdb.list

-in:file:fullatom

-ignore_unrecognized_res

-fa_max_dis 9.0

-database /home/usrrosetta/rosetta3.3_bundles/rosetta_database

-ddg::harmonic_ca_tether 0.5

-score:weights standard

-ddg::constraint_weight 1.0

-ddg::sc_min_only false

-score:patch /home/usrrosetta/rosetta3.3_bundles/rosetta_database/scoring/weights/score12.wts_patch

-ddg:out_pdb_prefix cst

-trust_missing_coords true

DDG generates the mutants’ structural models and calculates the Rosetta energy difference between the wild type protein and the mutants. Fixed backbone (low or mid-level precision) or modest backbone movement (high precision) modeling is carried out depending on the precision setup. Distance restraints can be considered in order to regulate the backbone movement within a certain range comparing to the crystal structures. A sample set of parameters for DDG_monomer calculation with mid-level precision is listed below:

-ddg:weight_file soft_rep_design # Use soft-repulsive weights for the initial sidechain optimization stage

-database /home/usrrosetta/rosetta3.3_bundles/rosetta_database #the full oath to the database is required

-fa_max_dis 9.0 # optional -- if not given, the default value of 9.0 Angstroms is used.

-ddg::iterations 50 # 50 is the recommended number of iterations

-ddg::dump_pdbs true # write out PDB files for the structures, one for the wildtype and one for the pointmutant for each iteration

-ignore_unrecognized_res # optional -- if there are residues in the input PDB file that Rosetta cannot recognize, ignore them instead of quitting with an error message

-ddg::local_opt_only false # recommended: local optimization restricts the sidechain optimization to only the 8 A neighborhood of the mutation (equivalent to row 13)

-ddg::min_cst true # use distance restraints (aka constraints) during the backbone minimization phase

-constraints::cst_file $PATH/min_cst_test.cst # the set of constraints to use during minimization which should reflect distances in the original (non-pre-relaxed) structure

-ddg::suppress_checkpointing true # don't checkpoint LIZ DOES CHECKPOINTING WORK AT ALL?

-in::file::fullatom # read the input PDB file as a fullatom structure

-ddg::mean false # do not report the mean energy

-ddg::min true # report the minimum energy

-ddg::ramp_repulsive true # perform three rounds of minimization (and not just the default 1 round) where the weight on the repulsive term is increased from 10% to 33% to 100%

-unmute core.optimization.LineMinimizer # optional -- unsilence a particular tracer

-ddg::output_silent true # write output to a silent file
